# The Clinical, Histological, and Genetic Spectrum of *RYR1* Variants—A Multi-Center Israeli Cohort Study

**DOI:** 10.3390/jcm15041388

**Published:** 2026-02-10

**Authors:** Mira Ginsberg, Marina Michelson, Sharon Aharoni, Liora Sagie, Yael Michaeli, Ditza Rotenberg, Vitaly Finkelshtein, Keren Yosovich, Zohar Argov, Andrea Nissenkorn, Dorit Lev, Menachem Sadeh, Ron Dabby

**Affiliations:** 1 Pediatric Neuromuscular Clinic, Pediatric Neurology Unit, Wolfson Medical Center, Holon 5822012, Israel; miraginsberg@gmail.com (M.G.); mashakerman@gmail.com (M.M.); 2 Gray Faculty of Medical and Health Sciences, Tel Aviv University, Tel Aviv 6997801, Israel; sharonah@clalit.org.il (S.A.); andrean@wmc.gov.il (A.N.); dorit.lev@gmail.com (D.L.); mesadeh@tauex.tau.ac.il (M.S.); 3 Pediatric Neurology Unit, Schneider Children Medical Center, Petch-Tikva 49202, Israel; 4 Pediatric Neurology Institute, Dana-Dwek Children’s Hospital, Tel Aviv Sourasky Medical Center, Tel Aviv 6423906, Israel; liorasag@gmail.com; 5 Pediatric Neurology Unit, Wolfson Medical Center, Holon 5822012, Israel; yaelyosef@gmail.com; 6 Independent Researcher, Ness-Ziona 740511, Israel; ditzarotenberg@gmail.com; 7 Department of Neurology, Wolfson Medical Center, Holon 5822012, Israel; 8 Institute of Medical Genetics, Wolfson Medical Center, Holon 5822012, Israel; kereny@wmc.gov.il; 9 Hadassa Medical Center, Jerusalem 9112001, Israel; zohara@ekmd.huji.ac.il; 10 Faculty of Medicine, Hebrew University, Jerusalem 9112102, Israel

**Keywords:** *RYR1* variants, King–Denborough syndrome, malignant hyperthermia, congenital myopathy

## Abstract

**Background**: Variants in the ryanodine receptor 1 (*RYR1*) gene have been linked to a range of disorders, from congenital myopathy to adult-onset manifestations, with phenotypes varying from mild to severe. **Methods**: A retrospective review was conducted on an Israeli cohort of 36 individuals with *RYR1* variants, identified through genetic testing as part of a national collaboration among multiple pediatric and adult neuromuscular clinics. Clinical features, molecular data, laboratory results, electromyographic findings, and muscle histology were analyzed. Each variant was classified according to its respective domain within the *RYR1* gene. **Results**: Thirty-six cases were included in the analysis; 31 were from 11 unrelated families, and 5 were sporadic. Nine individuals were asymptomatic with normal CK levels. Most of the 27 affected patients presented with variable degrees of perinatal weakness, often accompanied by respiratory impairment or arthrogryposis. Weakness was predominantly proximal, with clinical courses that included deterioration, improvement, or stabilization. Three cases of King–Denborough syndrome were identified. Additional presentations included malignant hyperthermia and, in isolated cases, periodic paralysis. Muscle biopsies demonstrated considerable histologic heterogeneity, including fiber-size variation, internal nuclei, multiminicores, and fibrosis or dystrophic features. The pathogenic *RYR1* variants included five compound-heterozygous genotypes, two homozygous variants, and two heterozygous variants. There was a positive correlation between variants located in the Bsol domain and disease severity. **Conclusions**: This cohort confirms and expands the clinical and histological diversity associated with *RYR1* variants in Israel. Variants in the Bsol domain appear to be indicative of disease severity.

## 1. Introduction

Ryanodine receptor 1 (RYR1) is a homotetrameric sarcoplasmic reticulum protein that functions as a channel releasing Ca^2+^ from intracellular stores. This process is triggered by conformational changes in the dihydropyridine receptor (DHPR), secondary to activation of acetylcholine receptors at the neuromuscular junction [1,2]. Thus, RYR1 plays a vital role in excitation–contraction coupling [3].

Variants in the *RYR1* gene (MIM #180901), which encodes the RYR1 protein, are the most prevalent cause of congenital myopathies. RYR1-related myopathies represent a heterogeneous group of autosomal recessive or autosomal dominant disorders, characterized by variable clinical presentations, diverse disease courses, and a wide range of histologic findings. The phenotypic spectrum includes congenital severe myopathy, which may occasionally show spontaneous improvement; syndromic myopathy such as King–Denborough syndrome (KDS) (a rare genetic disorder characterized by congenital myopathy, dysmorphic facial features, skeletal abnormalities, and a high susceptibility to malignant hyperthermia); myalgia; and malignant hyperthermia (MH) [4,5]. Moreover, with the advent of next-generation sequencing, additional conditions associated with pathogenic *RYR1* variants have been identified, including lethal multiple pterygium syndrome and periodic paralysis (PP) [6,7].

The most common histopathologic features of RYR1-related myopathies are central core disease (CCD) and multiminicore disease (MmD) [8]. However, other findings—such as nemaline rods (NRs), centronuclear myopathy (CNM), congenital fiber-type disproportion (CFTD), and dystrophic changes—have also been reported [9]. As in other myopathies, similar genetic variants, whether dominant or recessive, may present with diverse clinical manifestations, even within the same family [10,11]. Furthermore, clinical overlap exists between phenotypes caused by variants in RYR1 and other genes; for example, MH may also result from variants in CACNA1S [12,13].

The RYR1 complex is divided into several domains, including the N-terminal domain (residues 1–627), SPRY1 (residues 628–849), RY1 and RY2 (residues 850–1054), SPRY2 (residues 1055–1241), SPRY3 (residues 1242–1656), the junctional solenoid (Jsol; residues 1657–2144), and the bridging solenoid (Bsol) [14].

The aim of this study is to characterize the clinical, histopathologic, and electrophysiological findings, seeking correlations with the genetic heterogeneity and localization of the pathogenic variant of RYR1-related disorders in Israel.

## 2. Methods

### 2.1. Patients

The clinical, pathologic, and genetic findings obtained from Israeli individuals with *RYR1* variants were retrospectively analyzed. This cohort of patients was gathered through a national collaboration among multiple pediatric and adult neuromuscular clinics. The study was approved by the ethics committee of Wolfson Medical Center, Holon Israel. The cohort comprised 5 sporadic cases, 11 families with recessive inheritance, and 2 families with dominant inheritance. The clinical assessment included data regarding medical and family history, age of onset, initial presentation, muscle weakness distribution, skeletal deformities, dysmorphic features, and the clinical course. Laboratory evaluation included serum creatine kinase (CK) levels. HyperCKemia was defined as a CK level greater than twice the normal upper limit. Results of electromyography, brain MRI, and cardiorespiratory screening were also collected.

The severity of the disease was rated according to the following scale: 0—no symptoms; 1—myalgia and/or fatigability and/or skeletal deformity not including scoliosis; 2—mild weakness; 3—moderate weakness; 4—severe weakness with ambulation still possible and/or scoliosis; 5—wheelchair-bound; 6—wheelchair and respiratory support required.

### 2.2. Pathologic Assessment

Muscle biopsy samples were frozen in isopentane chilled in liquid nitrogen. Seven-micrometer-thick transverse sections were cut and stained with hematoxylin and eosin (H&E); modified Gomori Trichome; PAS; Oil Red O; NADH–Tetrazolium reductase; cytochrome oxidase with succinate dehydrogenase; ATPase at PH 9.4 and after preincubation at PH 4.3 and 4.6; and Congo red. Immunohistochemical staining for dystrophin 1–3; dysferlin; alfa-, beta-, gamma-, and delta-sarcoglycan; merosin; and caveolin 3 (all from Novoscastra Lab, Newcastle, UK) was performed using a Ventana Nexes automatic stainer—Roche diagnostics Mannheim Deutschland Gmbh (Mannheim, Germany). Staining was based on an indirect biotin–avidin system, resulting in dark-red precipitates at the antigens sites.

### 2.3. Evaluation of RYR1 Variants

*RYR1* variants were identified via exome or genome sequencing. Segregation in the families was validated via Sanger sequencing. The variants were classified according to the American College of Medical Genetics and Genomics (ACMG) guidelines [15]. Western blot analysis and quantification of RYR1 protein extracted from muscle biopsies were performed for one family only, using routine methods.

## 3. Results

### 3.1. Characterization of the Patients

Thirty-six pediatric and adult patients with homozygous or heterozygous (inherited or de novo) *RYR1* variants were assessed. The cohort consisted of 31 familial cases from 11 unrelated families and 5 unrelated sporadic cases. Two families (F-A and F-B) had been previously investigated [16,17]. Genetic data, clinical features, muscle morphology, and electrodiagnostic findings are summarized in Table 1 and Table 2 (families) and Table 3 (sporadic cases). In Table 1 and Table 2, only a single patient from families H, I, and J was included, as the other family members were not examined by our team. The familial cohort consisted of 24 affected and 8 unaffected individuals. Cases defined as affected included both symptomatic patients and those with asymptomatic hyperCKemia. In addition, seven carriers were identified through familial segregation analysis. The clinical, histopathological, and electrophysiological data refer only to the affected individuals. Among the affected familial patients, no difference in clinical severity scores was observed between males (*n* = 11) and females (*n* = 13). All familial and sporadic patients except one (P17) presented with muscle weakness at birth or during early infancy, often accompanied by delayed motor milestones. Decreased fetal movement suggesting antenatal involvement was not reported. The patients from families A, C, F, and I exhibited a progressive clinical course, whereas those from families E, G, and J had a stable course. Patients from families B, H, and K showed a benign course with noticeable improvement over the years. In family D, one patient experienced progression, while another remained stable. Most patients had proximal limb muscle weakness: 10 (34%) had lower-limb involvement, and 4 (14%) had both lower- and upper-limb involvement. Distal limb involvement was noted in seven (24%) patients; in three (10%) of them, it was accompanied by axial weakness. Neck muscles were affected in seven (24%) patients from birth or early infancy. Only three (10%) patients reported myalgia in adulthood, and in one patient (P11) it was the only clinical manifestation. Facial weakness was present in seven (24%) patients, with eye closure weakness in three, ophthalmoplegia in three, and ptosis in one. One patient experienced periodic paralysis attacks without weakness between episodes. Respiratory function was impaired in eight patients (28%): two (7%) had recurrent pneumonia in early childhood, and three (10%) developed gradual respiratory deterioration requiring nocturnal respiratory support. All affected members of family B required invasive ventilation during the neonatal period but were successfully weaned later in life. In family C, one patient exhibited ophthalmoplegia, while another presented with a rigid spine.

**Figure 1:**
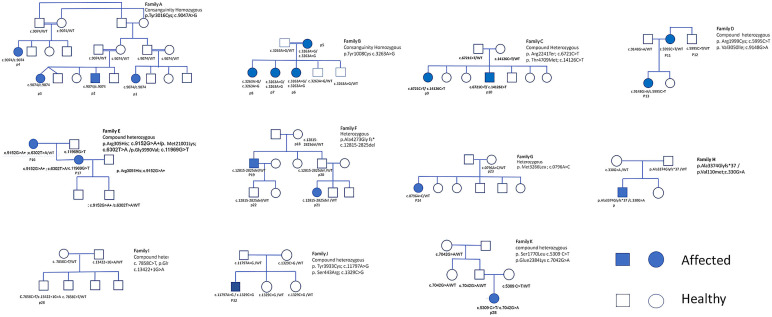
Pedigrees.

No feeding or swallowing difficulties were noted, and no cardiac involvement was observed. Three patients (P16, P19, and P36) had additional clinical features consistent with KDS (e.g., short stature and facial dysmorphism). Two patients (P5 and P16) experienced MH during surgery, the latter of whom was diagnosed with KDS. Mobility was preserved in most patients, regardless of their genetic variant. Serum CK levels were within the normal range for most patients, except for five (17%) who exhibited mild to moderate elevations. The familial pedigrees are presented in Figure 1.

### 3.2. Histopathological Findings

**Figure 2:**
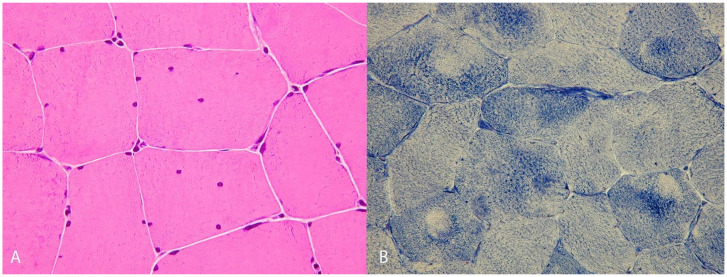
Characterization of the histological features of the muscle biopsy of P5: (**A**) H&E staining revealed an increased number of internal nuclei. (**B**) NADH staining shows areas of decreased enzymatic activity in type I fibers.

Muscle biopsies were performed on 18 patients. All showed non-specific myopathic changes, including variability in fiber size and internal nuclei. In two cases, NADH staining showed areas of decreased enzymatic activity in type I fibers (Figure 2). In five biopsies, mild endomysial fibrosis was also observed. In two biopsies, electron microscopy revealed multiminicores. Neither vacuoles nor abnormal protein aggregates were detected.

### 3.3. Molecular and Genetic Studies

In this cohort of 36 individuals, 23 distinct variants were identified, comprising 19 missense variants, one duplication (P25), and one intronic/splice-site variant (P26). Three nonsense variants resulted in premature stop codons: one caused by a single amino acid substitution (P9, P10, and P26), and another arising from a frameshift mutation (P18–P22).

A homozygous variant was detected in two inbred families: c.9047A>G, p.Tyr3016Cys in FA and c.3263A>G, p.Tyr1088Cys in FB, both of which showed severe neonatal presentations followed by significant improvement over time.

Variants in the Bsol domain were detected in 11 patients, either as homozygous variants in the four affected members of FA or as heterozygous variants in six familial cases (FC: P9, P10; FD: P13; FH: P25; FI: P26; FJ: P27; FK: P28) and one sporadic case (P34).

In eight patients, variants were identified in a compound heterozygous state, involving seven familial cases (FC-P9 and P10; FD-P13; FE-P16; FH-P25; FI-P26; FJ-P27; FK-P28) and one sporadic case (P34). Among the familial cases, individuals carrying a variant in the Bsol domain in combination with an additional variant exhibited a more rapidly progressive disease course and more severe myopathy—often accompanied by respiratory involvement and/or scoliosis (P9, P10, P13, P26, P25, and P28)—compared with those who carried a single variant located outside the Bsol domain, some of whom had only mild symptoms or remained asymptomatic. Similarly, the sporadic case P34 demonstrated a progressive course with scoliosis and respiratory difficulties.

## 4. Discussion

The RYR1 protein is located on the membrane of the sarcoplasmic reticulum within muscle cells and plays a pivotal role in regulating intracellular calcium by mediating its release from sarcoplasmic stores. The released calcium ions bind to troponin C, inducing a conformational change in the troponin complex. This, in turn, triggers the formation of cross-bridges between the contractile proteins actin and myosin, enabling their sliding interaction and ultimately generating muscle contraction [27,28].

**Figure 3:**
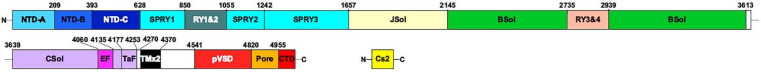
Two-Two-dimensional representations of the domain boundaries of the *RYR1* monomer described in the work of des Georges et al. [14] (obtained with permission).

The emergence of high-resolution cryo-electron microscopy (cryo-EM) and X-ray crystallography has provided valuable insights into the structure and function of RYR1. By resolving its three-dimensional architecture, these techniques have enabled researchers to better understand both normal and mutated conformations of the protein [29]. RYR1 is composed of four subunits, each containing multiple functional domains, and is divided into two major components: a cytosolic shell formed by the N-terminal domains, and a channel and activation core comprising the remaining C-terminal segments. Bridging these components are the solenoid structures from each protomer, known as the junctional solenoid (JSol) and the bridging solenoid (BSol) [14] (Figure 3).

The BSol domain plays a central role in the conformational transitions between the open and closed states of the RYR1 channel through its interactions with other domains, including Nsol. Variants in the BSol domain can disrupt the structural conformation of the RyR1 channel and impair calcium homeostasis. Such disruption may result in pathological calcium release, either through leakage due to incomplete channel closure or through insufficient opening, leading to reduced calcium release and consequent muscle weakness and myopathy. Indeed, studies have shown that biallelic variants in the BSol domain are frequently associated with severe clinical phenotypes, including marked muscle weakness, respiratory insufficiency, and feeding difficulties during infancy [1,2].

These observations were also confirmed in our cohort, in which most patients who were wheelchair-bound or exhibited a severe progressive course were found to carry at least one variant in the BSol domain. Notably, in Family F and in a sporadic case (P34), the variant p.Arg1999Cys (c.5995C>T), when present in combination with another variant, was associated with a severe and progressive disease trajectory.

Our series encompassed a heterogeneous range of clinical presentations. Most patients exhibited a phenotype characterized by neonatal or early-onset progressive muscle weakness. In the most severe cases, the disease progressed to the point of requiring wheelchair use and mechanical ventilation. Conversely, at the milder end of the spectrum, some patients demonstrated a disease course marked by clinical improvement. Across this variability, muscular symptoms such as myalgia and muscle weakness were common, and a subset of patients experienced malignant hyperthermia (Table 1, Table 2 and Table 3).

The high prevalence of severe cases in our series may be somewhat biased, as milder presentations could be underdiagnosed. Notably, five asymptomatic individuals with normal CK levels carried heterozygous variants, similar to their affected family members. Reduced penetrance and clinical heterogeneity of *RYR1*-related disorders have been observed in other studies and are suggested to result from exposure to external factors, including physical exercise, heat, fever, or polymorphisms that modulate calcium homeostasis [30]. The existence of asymptomatic and mildly symptomatic individuals with *RYR1* variants complicates the interpretation of genetic testing. Functional studies, when available, may help confirm the pathogenicity of the variant.

The most frequent pathological finding in muscle biopsies of patients with *RYR1* variants is the presence of central cores, which have been particularly associated with dominant inheritance [31]. Other pathological findings, such as multiminicores [32], centronuclear myopathy [33], and fiber-type disproportion [9], have been primarily linked to recessive disease. However, in our cohort, most biopsies demonstrated non-specific myopathic changes, including fiber size variation, internal nuclei, endomysial fibrosis, and type 1 fiber predominance. Only three biopsies from familial patients showed multiminicores (P9, P27, and P28), while one biopsy from a sporadic case revealed fiber-type disproportion (P34). The absence of typical *RYR1*-related myopathy features in most biopsies underscores that muscle biopsy cannot reliably diagnose *RYR1*-related conditions, particularly when genetic studies identify variants of uncertain significance. Genetic testing is especially valuable when muscle biopsy findings are inconclusive or normal, as observed in atypical presentations such as rhabdomyolysis, myalgia, or periodic paralysis. Nonetheless, interpretation of genetic results should be integrated with clinical, histological, and imaging data to achieve an optimal and accurate diagnosis.

Two of our patients experienced malignant hyperthermia (MH). One patient (P5) had been previously reported in a Samaritan family with benign congenital myopathy [17], while the other (P16) carried a newly identified variant that has not been previously described. To date, 29 different variants have been associated with MH [30,34], most of which are located within known hotspot regions of *RYR1* [30]. However, the two variants identified in our cohort were situated outside these hotspot domains.

Two familial patients and one sporadic patient presented with KDS (P16, P19, and P36). All exhibited dysmorphic features, short stature, skeletal abnormalities, and myopathy. One patient had MH susceptibility confirmed via a caffeine test. Muscle biopsy findings varied and included fiber size variation, internal nuclei, fat and connective tissue replacement, and dystrophic features. Central cores were not observed. One patient (P16) carried three heterozygous missense variants, one (P19) carried a heterozygous nonsense variant, and one (P36) harbored a de novo heterozygous missense variant. None of these variants were located in the Bsol domain.

In family F, all members carried the c.12815_12825, p.Ala4272Glyfs*307 variant, yet one member exhibited a mild phenotype while three others were asymptomatic, despite carrying the same pathogenic variant. Similar discrepancies, where the proband with KDS harbored a heterozygous missense variant, while other family members with the same variant displayed only mild symptoms or were clinically asymptomatic, have been reported [35,36].

Phenotypic variability in *RYR1* patients results from the combined effects of variant type, inheritance pattern, affected protein domains, environmental factors, modifier genes, and post-translational modifications. Single heterozygous variants are often linked to milder phenotypes like central core disease and malignant hyperthermia susceptibility [10]. In contrast, biallelic variants have been associated with early onset generalized weakness, respiratory insufficiency, and multisystem involvement [11]. Compound heterozygosity and the location of variants beyond well-defined regions increased phenotypic complexity [11]. Environmental factors, modifier genes, and post translational changes to myosin further contribute to the variability [37].

The reason for the phenotypic variability among patients within the same family, including those with KDS, remains unclear. Proposed explanations include the presence of a genetic modifier that may influence the expression of the unaffected *RYR1* allele and the preferential expression of the non-mutated allele in asymptomatic or mildly symptomatic family members [35].

Similar to our findings, recent large cohort studies of *RYR1*-related myopathies from England and Italy report considerable clinical heterogeneity, with phenotypes ranging from asymptomatic or mildly affected individuals (including isolated hyperCKemia) to patients with severe muscle weakness, loss of ambulation, skeletal deformities, and significant respiratory involvement [8,38]. In accordance with our cohort, severe phenotypes were more frequently observed in patients with early disease onset, typically at birth, during infancy, or in early childhood. Furthermore, most severe cases were associated with autosomal recessive inheritance, predominantly involving homozygous or compound heterozygous pathogenic variants. These observations support an association between biallelic pathogenic variants and greater disease severity, although genotype–phenotype correlations remain variable.

## 5. Conclusions

The presented data expands our knowledge about the clinical, histological, and genetic spectrum of *RYR1* variants in Israel and suggests that the range of variants and clinical presentations is broader than previously recognized. Variants in the Bsol domain are characterized by greater disease severity, suggesting that this region may serve as a potential target for therapeutic intervention.

## Figures and Tables

**Table 1:** Genetic characteristics of families with *RYR1* variants. Variants classification is according to ACMG criteria.

	Segregation Affected/Unaffected	Genetic Variation NM_000540.3	Domain	Onset	Current Age/ Gender	Reference
**Family A**	**4 affected (P1–P4)** Homozygous Consanguinity	**P1–P4**: c.9047A>G; p.Tyr3016Cys Missense, P (PS4, PP1, PS3, PM2, PP3, PP2) Clinvar ID RCV000558544	BSol	**P1**: neonatal **P2**: 1.5 Y **P3**: 6 Y **P4**: 4 Y	**P1**: 38 Y/F **P2**: 25 Y/M **P3**: 18 Y/M **P4**: 17 Y/M	[16]
**Family B**	**4 affected (P5–P8)** Homozygous Consanguinity	**P5–P8**: c.3263A>G; p.Tyr1088Cys Missense, LP (PM2, PP3, PP2, PP5) UniProt ID VAR_068512	SPRY2	**P5–P8**: neonatal	**P5**: 43 Y/F **P6**: 21 Y/F **P7**: 21 Y/F **P8**: 17 Y/F	[17]
**Family C**	**2 affected (P9, P10)** Compound heterozygous	**P9, P10**: c.6721C>T; p. Arg2241Ter Nonsense, P (PM3, PS3, PVS1, PM2) ClinVar ID RCV000147436 c.14126C>T; p.Thr4709Met Missense, LP (PM2, PM5, PP3PP2, PP5) ClinVar ID RCV000119498	**I.** BSol **II**. pVSD	**P9, P10**: neonatal	**P9**: 15 Y/M **P10**: 22 Y/F	[18]
**Family D**	**2 affected (P11, P13)** 1 Heterozygous 1 Compound heterozygous **1 unaffected** (**P12**) Heterozygous	**P11, P13**: c.5995C>T; p.Arg1999Cys Missense, LP (PM2, PP3, PP2, PM3) ClinVar ID RCV001047012 **P12, P13** c.9148G>A; p.Val3050lle Missense, VUS (PM2, PM5, PP2, BP6 PP1) ClinVar ID RCV001660679	**I**. JSol II. BSol	**P11**: 10 Y **P13**: neonatal	**P11**: 66 Y/F **P12**: 68 Y/M **P13**: 40 Y/F	
**Family E**	**2 affected**: **1** heterozygous (2 mutations **P17**) ** 1 Compound heterozygous (3 mutations-**P16**) **2 unaffected** 1 heterozygous (1 mutation -**P14**) 1 heterozygous (2 mutation-**P15**) **	**P15–P17**: c.9152G>A; p. Arg3051His Missense, LP (PM2, PP2, PM3) ClinVar ID RCV001803928 c.6302T>A; p. Met2101Lys Missense, VUS(PM2, PP2, PM3) ClinVar ID RCV001592839 **P14, P16** c.11969G>T; p.Gly3990Val Missense, LP (PP1, PS3, PM2, PP3, PP2, PP5 PM3) ClinVar ID RCV002281939	**I.** NTD-B **II**. JSol **III.** CSol	**P14, P15**: asymptomatic **P16**: 15 Y **P17**: 4 Y	**P14**: 69 Y/M **P15**: 66 Y/F **P16**: 31 Y/F **P17**: 4 Y/M	III [19]
**Family F**	**2 affected** (**P19, P21**) Heterozygous **3 unaffected** (**P18, P20, P22**) Heterozygous	**P18–22**: c.12815_12825del p.Ala4272Glyfs*307, c.12815_12825del NM_000540.3 LP/LP Nonsense, LP (PVs1, PM2)	TaF	**P19**: neonatal **P21**: 1.5 Y **P18, P20, P22;** asymptomatic	**P18**: 70 Y/F **P19**: 45 Y/M **P20**: 39 Y/M **P21**: 17 Y/F **P22**: 16 Y/M	
**Family G**	**1 affected** (**P24**) Heterozygous **1 unaffected** (**P23**) Heterozygous	P23, P24: c.9796A>C; p. Met3266Leu Missense, VUS (PM2, PP2) ClinVar ID RCV001368477	BSol	**P24**: 14.5 Y	**P23**: 46 Y/F **P24**: 16 Y/F	[20]
**Family H**	**1 affected (P25)** Compound heterozygous	**P 25**: c.11320dup; p. Ala3374Gly fs*37 (PS4, PVS1, PM2) ClinVar ID RCV000721230 c.3301G>A; p. Val1101Met Missense LP PM2, PP3, PP2, PP5 ClinVar ID RCV001852321	**I** BSol **II** NTD-A	**P25**: neonatal	**P25**: 1.5 Y/M	
**Family I**	**1 affected (P26)** Compound heterozygous	P26: c.13437+1G>A Splicing, LP (PVS1, PM2, PP5) ClinVar ID RCV000721317 c.7858C>T; p.Gln2620Ter Nonsense, P (PS4, PVS1, PM2, PM3) ClinVar RCV001219907	**I.** BSol **II**.SPRY2	**P26**: neonatal	**P26**: 37 Y/M	
**Family J**	**1 affected (P27)** Compound heterozygous	**P27**: c.11798A>G; p.Tyr3933Cys Missense, LP (PP3, PM2, PP2) ClinVar ID RCV000148797 c.1329C>G; p. Ser443Arg Missense, LP (PP1, PM2, PP2, PM3)	**I** Nsol **II** CSol	**P27**: Neonatal	**P27**: 15.5 Y/M	[21]
**Family K**	**4 affected (P28–P31)** 1 Compound heterozygous 3 Heterozygote	**P28**: c.3509C>T; p. Ser1770Leu; Missense, LP (PM2, PP2, PM3, PP1) ClinVar ID RCV000079149 **P28–P31** c.7042G>A; p. Glu2348Lys Missense, LP (PM1, PP2, PM2, PM5, PP3) ClinVar ID RCV000721635	**I**. Bsol **II**. Jsol	**P28**: Neonatal	**P28**: 7.5 Y/F **P29**: M **P30**: F	[22]

ACMG—American College of Medical Genetics and Genomics: P pathogenic variant, LP likely pathogenic. ** Two heterozygous variants located on the same allele.

**Table 2:** Clinical and ancillary characteristics of familial patients with *RYR1* variants.

	Muscle Involvement	Facial Involvement	Respiratory Involvement	Skeletal Involvement	CK Levels	EMG	Biopsy Findings	Severity	Motor Development/Course
**Family A** **(P1–P4)**	**P1**–**P4**: neck and limb girdle muscle weakness **P1**: wheelchair bound since 11 Y	**P1**–**P4**: facial muscles and eye closure weakness, dysmorphic features: elongated face	**P1**: recurrent severe pneumonia	**P1**: scoliosis	**P1**: mildly elevated **P2, P3, P4**: normal	**P1**: myopathic changes	**P1–P4;** Fiber size variation, internalized and central nuclei, endomysial fibrosis	**P1**: 5 **P2**: 5 **P3**: 4 **P4**: 4	**P1**: rapid progression since birth **P2–P4**: variable progressive
**Family B** **(P5–P8)**	**P5–P8**: congenital hypotonia &weakness, by 2 Y improved to have only distal limbs weakness **P5**: MH during surgery	**P5–P8**: facial weakness, bi-temporal Narrowing, epicanthal folds, hypertelorism	**P5**–**P8**: neonatal, improved gradually within 2 weeks		**P5–P7**: normal **P8**: NA	**P5**: myopathic changes	**P5**: Internalized and central nuclei, central areas devoid of oxidative enzyme activity and moth-eaten appearance	**P5**: 1 **P6**: 1 **P7**: 1 **P8**: 1	**P5–P8**: delayed/Improving
**Family C** **(P9, P10)**	**P9, P10**: congenital hypotonia, neck and limb weakness, **P9**: never walked and wheelchair bound **P10**: wheelchair bound since age 7 Y	**P9**: ophthalmoplegia	**P9**: night BI-PAP since 7 y **P10**: neonatal respiratory weakness, needing ventilation for 4 days, night BI-PAP since 12 Y	**P9**: scoliosis& rigid spine, operated at 15 y **P10**: scoliosis, surgery pending	**P9, P10**: normal	**P9, P10**: ND	**P9**: **LM**: great variability in fiber size **EM**: minicores and large mitochondria	**P9**: 6 **P10**: 5	**P9**: delayed/progressive **P10**: severely delayed/progressive
**Family D** **(P11–P13**)	**P11**: myalgia and fatigability since early age **P13**: congenital proximal muscle weakness, wheelchair bound since 13 Y	**P13**: eye closure weakness	No	**P13**: scoliosis	**P11, P13**: normal	**P11**: normal **P13**: myopathic changes-severe	**P13**: type 1 fiber predominant, atrophy and grouping	**P11**: 1 **P12**: 0 **P13**: 5	**P11**: delayed/stable **P13**: Delayed/ progressive:
**Family E** **(P14–P17)**	**P16**: mild weakness, MH during surgery	**P16**: KDS: dysmorphic face, high arch palate	No	**P15**: short stature **P16**: mild scoliosis, joints deformity, pes cavus	**P14, P15**: NA **P16**: moderately elevated **P17**: mildly elevated	**P15**: normal **P14–p17**: ND	**P16**: fiber size variation internal nuclei positive findings with caffeine exposure	**P14**: 0 **P15**: 1 **P16**: 0	**P16**: normal/stable
**Family F** **(P18–P22)**	**P19**: proximal and distal lower &upper limbs weakness **P21**: proximal lower limbs weakness	**P19**: KDS: dysmorphic faces **P21**: weakness eye closure	No	**P19**: short stature, arthrogryposis	**P19**: NA **P21**: normal	**P19**: severe myopathic feature **P21**: normal	**P19**: fiber size variation, internal nuclei. perimysium replaced by fat and connective tissue **P21**: myopathic features	**P19: 5** **P21: 2**	**P19**: severely delayed/progressive **P21**: mildly delayed/ slowly progressive
**Family G** **(P23, P24)**	Normal	No	No	**P24**; episodic weakness	Normal	**P24**: normal	ND	**P24**: variable	**P24**: normal/stable
**Family H** **(P25)**	General hypertonia, poor sucking and crying, no head control	No	C-PAP night ventilation since 20 y	No	Mildly elevated	ND	ND	4	**P25**: mildly delayed/Improving
**Family I** **(P26)**	Proximal and distal lower limbs weakness, muscle atrophy, wheelchair bound since age 7 Y	Limited upper gaze &ptosis		Joint contractions, scoliosis	Mildly elevated	Myopathic features	Fiber type variation and internal nuclei	−5	**P26**: severely delayed/progressive
**Family J** **(P27)**	Proximal weakness	No		No	Normal	ND	Fiber type variation and internal nuclei EM: multi mini- core	2	**P27**: severely delayed/stable
**Family K** **(P28–P30)**	**P28**: proximal weakness	No	No	**P28**: scoliosis operated	**P28**: Normal **P29–P30** moderate elevated	ND	**P28**: Multi mini core	2	**P28**: moderately delayed/Improving **P29–P31**: normal/Improving

CK Serum creatine kinase level; NA None available; EMG electromyography; ND Not done; EM electron microscopy; LM light microscopy, M malignant hyperthermia, KDS King–Denborough syndrome.

**Table 3:** Genetic and clinical characteristics of sporadic patients with *RYR1* variants.

Patient	P32	P33	P34	P35	P36
**Gender**	Male	Female	Female	Male	Male
**Current age/Onset**	66 Y/50 Y	10 Y/neonatal	35 Y/neonatal	20 Y/18 Y	26 Y/Neonatal
**Genetic variants** **Variant** **NM_000540.3**	Heterozygous c.528G>T; p. Glu176Asp missense, LP (PM2, PM1, PP2, PP4) ClinVar ID RCV001580415	* De novo heterozygous c.12083C>T; p. Ser4028Leu missense, P (PP1, PS3, PM2, PP) ClinVar ID RCV000721259	Compound heterozygous c.5995C>T; p.Arg1999Cys missense, LP (PM2, PP3, PP2, PM3) ClinVar ID RCV001047012 c.6721C>T nonsense, P p. Arg2241 * (PM3, PS3, PVS1, PM2) ClinVar ID RCV000147436	* De novo heterozygous c.15067T>C missense, LP p. Phe5023Leu; PP3, PM2, PP2) ClinVar ID RCV001225325	* De novo heterozygous c.14818G>A missense, LP p. Ala4940Thr; (PM1, PP2, PM2, PP3) ClinvarID RCV000119566
**Domain**	NTD-A	CSol	Bsol, JSol	CTD	S6c
**Muscle involvement**	Lower limbs muscle weakness, myalgia, atrophy of quadriceps and lumbar and upper thoracic muscles	Proximal lower limbs weakness delayed motor milestones	Proximal and distal lower limbs	Myalgia	Lower and upper limbs
**Respiratory involvement**	No	Recurrent pneumonia in early childhood	Respiratory difficulties	No	No
**Skeletal involvement**	No	No	Scoliosis/operated	No	KDS: scoliosis, Short stature
**CK levels**	1000–3000 **	normal	NA	800–100	NA
**EMG**	Myopathic features	ND	NA	NA	ND
**Biopsy findings**	Muscular dystrophy	Fiber size variation	Fiber type disproportion	ND	Dystrophic features
**Course**	progressive	stable	progressive	stable	progressive
**Severity**	4	2	6	1	5
**Reference**		[23]	[24]	[25]	[26]

ACMG—Variant classification is according to ACMG criteria, CKserum creatine kinase; NA—Not available; EMG—electromyography; ND—Not done. * Family segregation did not detect mutation in other family members. ** Other family members suffered from malignant hyperthermia.

## Data Availability

The data are not publicly available due to privacy and ethical restrictions related to the sensitive genetic and clinical information of the participant. All relevant data generated or analyzed during this study are included in this published article. Further inquiries can be directed to the corresponding author.
